# Contrast-Based Fully Automatic Segmentation of White Matter Hyperintensities: Method and Validation

**DOI:** 10.1371/journal.pone.0048953

**Published:** 2012-11-12

**Authors:** Thomas Samaille, Ludovic Fillon, Rémi Cuingnet, Eric Jouvent, Hugues Chabriat, Didier Dormont, Olivier Colliot, Marie Chupin

**Affiliations:** 1 Université Pierre et Marie Curie – Paris 6, Centre de Recherche de l'Institut du Cerveau et de la Moëlle Epinière, UMR-S975, Paris, France; 2 Inserm, U975, Paris, France; 3 CNRS, UMR 7225, Paris, France; 4 ICM – Institut du Cerveau et de la Moëlle epinière, Paris, France; 5 AP-HP, Pitié-Salpêtrière University Hospital, Department of Neuroradiology, Paris, France; 6 AP-HP, Lariboisière University Hospital, Department of Neurology, Paris, France; 7 Inserm, UMR 740, Paris, France; 8 DHU NeuroVasc Sorbonne Paris-Cité, Paris, France; University of Manchester, United Kingdom

## Abstract

White matter hyperintensities (WMH) on T2 or FLAIR sequences have been commonly observed on MR images of elderly people. They have been associated with various disorders and have been shown to be a strong risk factor for stroke and dementia. WMH studies usually required visual evaluation of WMH load or time-consuming manual delineation. This paper introduced WHASA (White matter Hyperintensities Automated Segmentation Algorithm), a new method for automatically segmenting WMH from FLAIR and T1 images in multicentre studies. Contrary to previous approaches that were based on intensities, this method relied on contrast: non linear diffusion filtering alternated with watershed segmentation to obtain piecewise constant images with increased contrast between WMH and surroundings tissues. WMH were then selected based on subject dependant automatically computed threshold and anatomical information. WHASA was evaluated on 67 patients from two studies, acquired on six different MRI scanners and displaying a wide range of lesion load. Accuracy of the segmentation was assessed through volume and spatial agreement measures with respect to manual segmentation; an intraclass correlation coefficient (ICC) of 0.96 and a mean similarity index (SI) of 0.72 were obtained. WHASA was compared to four other approaches: Freesurfer and a thresholding approach as unsupervised methods; k-nearest neighbours (kNN) and support vector machines (SVM) as supervised ones. For these latter, influence of the training set was also investigated. WHASA clearly outperformed both unsupervised methods, while performing at least as good as supervised approaches (ICC range: 0.87–0.91 for kNN; 0.89–0.94 for SVM. Mean SI: 0.63–0.71 for kNN, 0.67–0.72 for SVM), and did not need any training set.

## Introduction

With the increasing use of MRI in neuroimaging in the last 30 years, the detection of white matter (WM) lesions, appearing as white matter hyperintensities (WMH) on T2-weighted images, has become a common finding in elderly subjects. Its prevalence may reach up to 95% over 65 years old [1] with a steady progression of volume with age [2]. Although they are observed in both healthy and diseased subjects, numerous studies have established an association between WMH and stroke [3], late onset depression [4], Alzheimer's disease [5], impairment of gait [6], cognitive deficits [7] and risk of dementia [8]. A recent clinical meta-analysis [9] demonstrated that WMH increase risk of dementia, stroke and death and is a potential sign of cerebrovascular disorders which should require supplementary clinical investigation.

First pathological studies of WMH were undertaken in [10]; they concluded on a large spectrum of findings, such as demyelination, ependymitis, rarefaction of axons and gliosis. The aetiologies of WMH remain unclear but the strongest hypothesis is that they would be linked with small vessel disease. Thickening walls of small vessels would induce chronic hypoperfusion and disruption of the blood-brain barrier. More frequent and more extensive WMH in patients with cardiovascular risk factors support this hypothesis. Cerebral Autosomal Dominant Arteriopathy with Subcortical Infarcts and Leucoencephalopathy (CADASIL), a hereditary small vessel disease caused by mutations in the NOTCH3 gene [11], exhibits the same changes and became a model for studying the mechanisms of small vessel disease [12].

The diversity of underlying damages made the classification of WMH difficult and different visual rating scales were subsequently developed to grade their severity, depending on their location, size, shape and number. However, the heterogeneous properties of these scales resulted in inconsistencies between studies that prevented comparison of results [13] and pushed towards the development of quantitative methods, in which physicians manually outlined WMH [14] [15]. Accurate segmentation of WMH instead of subjective assessment of the total lesion load volume also enables finer analyses of the topography of WMH and their correlations with cognitive deficits. Duering et al [16] used a voxel-based lesion-symptom mapping approach based on manually edited segmentations on data from 215 patients with CADASIL and found an influence of lesion load in strategic anatomical sites, such as the anterior thalamic radiation, on a compound score for processing speed while there was no independent contribution of total volume of WMH.

However, manual outlining of WMH is time consuming and suffers from interrater variability. These aspects prevented the development of further studies of topography and regional volume of WMH through large databases. Automated segmentation methods have thus been proposed as a mean to make such analyses feasible and robust. They vary greatly in terms of complexity, computational time and required image modalities. They can be divided into unsupervised and supervised methods.

In the first category, Jack et al [17] proposed to segment WMH by using a simple threshold derived from a regression analysis on the histogram of the Fluid Attenuated Inversion Recovery (FLAIR) image. Wen and Sadchev [18] suggested a more robust way to compute this threshold based on statistics of WM intensities derived from a probabilistic atlas of WM and used information from T1 images to remove false positives. Admiraal-Behloul et al [19] reported good results when combining Proton Density (PD), T2 and FLAIR images with a probabilistic atlas of WM into a fuzzy inference system. Gibson et al [20] proposed to combine fuzzy C-means clustering with thresholding and evaluated two false positive minimization methods. Maillard et al [21] developed an algorithm based on multispectral classification from T1, T2 and PD images. They did not evaluate it with respect to manual segmentation but showed that its results correlated with visual scales. In recent work, Smith et al [22] customised the Freesurfer software for WMH segmentation, and reported high intraclass correlation on 10 subjects.

In the second category, each voxel is represented in a feature space that is constructed from segmentation-relevant characteristics derived from several images. Supervised methods learn from manually segmented data how to differentiate WMH voxels from intact voxels within this feature space and then classify each new voxel according to its location in this feature space with respect to training data. Anbeek et al [23] used k-nearest-neighbours (kNN) classification on T1, T2, Inversion Recovery (IR), PD and FLAIR images of 20 patients and reported good overlap measures, but their results were highly variable with respect to total lesion load. De Boer et al [24] proposed a similar approach by classifying normal tissues (GM, WM and CSF) with kNN and subsequently segment WMH by thresholding of the FLAIR histogram of voxels classified as GM. Thresholding parameters were optimized on a subset of six subjects and applied on images from 215 patients. However, quantitative evaluation was only provided for 20 subjects (including the six subjects of the training set) with a mean SI of 0.75 and all data were acquired on the same scanner. Support vector machine (SVM) is another supervised method that has been used by more recent studies [25] [26]. Lao et al [25] reported high correlation measurements between manual and automatic segmentation for 45 subjects but did not evaluate spatial agreement.

The above methods are difficult to directly compare, as they were evaluated on different datasets and with various quantitative indices. In recent work, Klöppel et al [26] evaluated three of these methods on the same dataset, composed of T1 and FLAIR images from 20 patients acquired in a single centre. They evaluated Otsu's thresholding [27] as unsupervised method, kNN and SVM as supervised ones. A framework for common preprocessing steps and identical learning sets was developed. The best results reported were obtained with SVM.

Although better results were reported with supervised methods on this small sample, these methods may face difficulties when used to segment subjects from new centres or with yet unseen pathological characteristics. The ideal learning set should embed the full range of variability that may occur in acquisition and pathology. Unfortunately, this condition is nearly impossible to achieve, and the method may perform very well for a subject with similar characteristics compared to the samples in the learning set, but very poorly for new subjects with dissimilar characteristics. This problem is known as overfitting.

To overcome these issues, we propose here a new method specifically designed for being robust to acquisition and pathological variation. The White matter Hyperintensities Automated Segmentation Algorithm (WHASA) method relies on contrasts rather than intensities; contrast is indeed less variable than intensity values with respect to acquisition. Evaluation of WHASA has been carried out on 67 patients exhibiting large lesion load variability and scanned on different MRI scanners; a full comparison study was undertaken with respect to other state-of-the-art unsupervised and supervised methods. We will first describe the WHASA method. Datasets and indices used for validation will then be described before introducing other methods selected for comparison. The performance of WHASA and the other methods on our dataset will then be presented and discussed.

## Methods

### Ethics statement

The protocol and informed consent forms were approved by the Ethics Committee of Salpêtrière Hospital for MCI patients (dataset 1) and by the Ethics Committee of Lariboisière Hospital for CADASIL patients (dataset 2). Participants had given their written informed consent.

### WHASA method

FLAIR images have been considered as more suitable for characterising WMH contrast and intensity properties for many years, as the signal from the cerebrospinal fluid (CSF) is nulled out and only grey matter (GM) and WMH remain brighter than WM, and are used in clinical routine for visual estimates. WMH visual detection depends on their contrast with respect to surrounding tissues as well as their location into the white matter. WHASA is based on these two characteristics. Standard preprocessing steps (section “Preprocessing”) extract tissue information from T1 images, register it to the FLAIR image and correct for intensity inhomogeneities. Non linear diffusion framework enables then to enhance contrast of WMH on the FLAIR image and obtain a piecewise constant image (section “Segmentation of the FLAIR image”). Finally, tissue information obtained from preprocessing steps allows the selection of relevant regions according to their location (section “Selection of segmented regions”). The voxels being highly anisotropic on clinical 2D FLAIR images (slice thickness about 5 mm), the segmentation of the FLAIR image and the selection of regions, both steps that rely mainly on the FLAIR image, were implemented in 2D.

#### Preprocessing

Three preprocessing steps using SPM8 software (http://www.fil.ion.ucl.ac.uk/spm/) were applied before segmenting WMH.

- Step 1: The New Segment module of SPM8 was applied on T1 images [28]. This combined tissue segmentation, spatial normalisation and image inhomogeneity correction approach resulted in probabilistic maps of gray matter (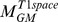), white matter (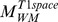), cerebrospinal fluid (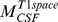), meninges (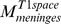) and skull (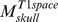) in T1 space as well as bias corrected T1-weighted image (*mT1*). The computed non-linear transformation was also applied to “back-register” the white matter MNI template to the T1 space (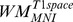) as well as the mid-sagittal plane (*msP^T1 space^*).
- Step 2: Rigid body transformation was computed to register *mT1* to the FLAIR image (“coreg” function of the SPM8 software). The resulting transformation was then applied to all the above mentioned images resulting in 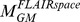, 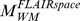, 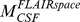, 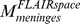, 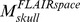, 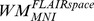, *mT1^FLAIR space^* and 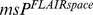. For notation simplicity, since the rest of the algorithm will take place in the FLAIR space, “*FLAIR space”* will be omitted in the remaining of the description of the methods.
- Step 3: FLAIR image was bias corrected using the multimodality mode of the New Segment function. It will be noted *mFLAIR* in the following.

The average computing time for these preprocessing steps was about 20 mins for each patient.

#### Segmentation of the FLAIR image

**Figure 1:**
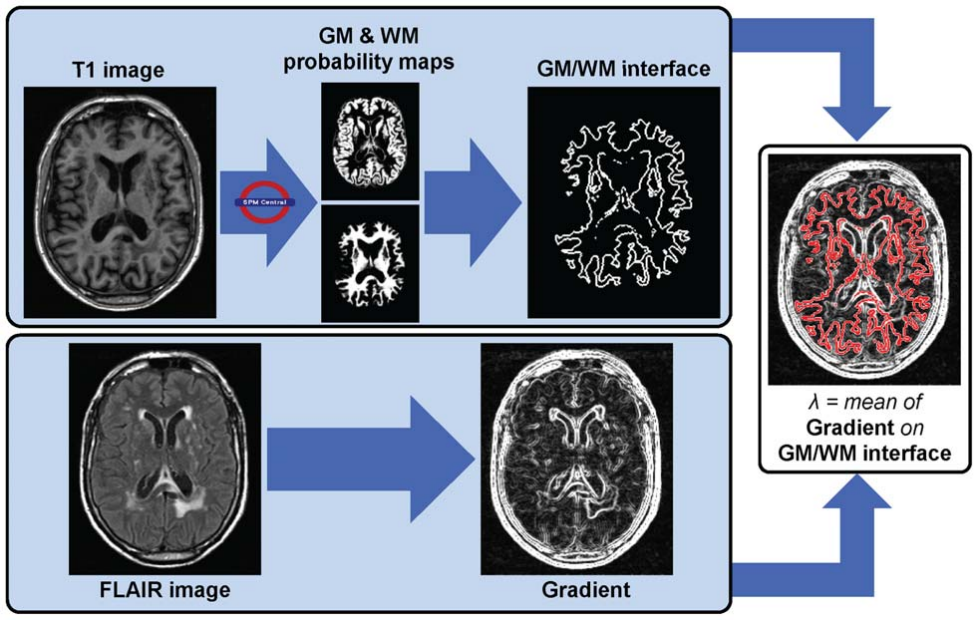
Computation of the contrast parameter λ for non linear diffusion.

**Figure 2:**
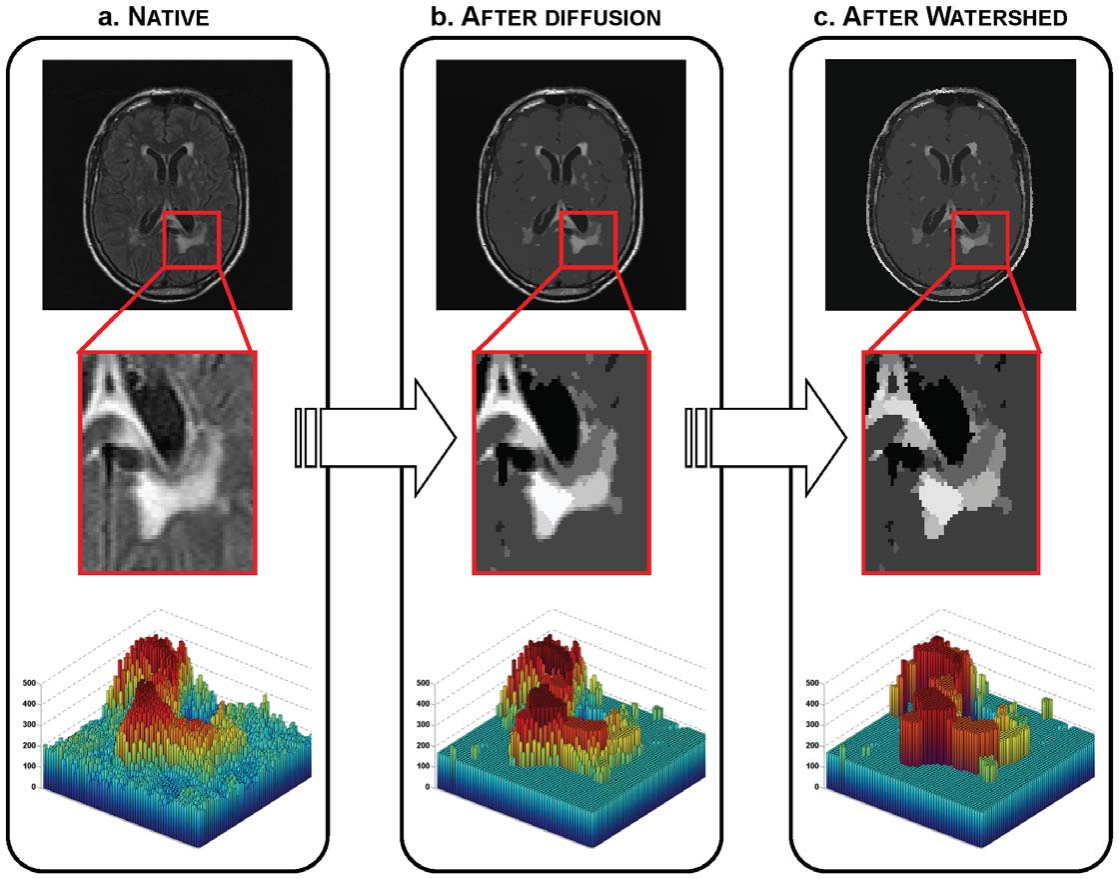
Illustration of the segmentation of the FLAIR image. First row: one slice of an image and its evolution through the algorithm. Second row: enlargement of the part in the red square. Third row: 3D visualization of the enlarged part where colour and height indicate intensity values. **a.** Original FLAIR image; **b.** Result of the non linear diffusion (last iteration); **c.** Piecewise constant image from watershed applied on the diffused image.

The segmentation process as detailed below is illustrated in Figure 1 and Figure 2.

Non linear diffusion was introduced in [29]. It enables spatial-dependent filtering based on the gradient of an image I.
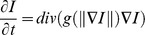
where *g* is a diffusivity function that decreases with respect to gradient magnitude. Different diffusivity functions have been studied in [30] and a robust *g* function was derived from Tukey's biweight function.
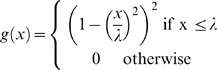



It depends on a contrast parameter λ, characterizing minimal contrast between regions. Contrary to other suggested *g* functions where diffusion stops only for a uniform image, this diffusion process reaches a steady state when no gradient magnitude is below λ.

This step was performed with the freely available *Non linear diffusion* MATLAB toolbox customised to include the Tukey's biweight function.

A critical parameter to set is thus the contrast parameter. In our case, WMH have to be differentiated mainly from surrounding white matter (WM) on FLAIR images. Non linear diffusion was applied to *mFLAIR* to enhance edges between WM and WMH while weakening edges between WM and GM. The contrast parameter λ was thus set to the mean contrast between WM and GM (Figure 1).

To define the interface between GM and WM, *M_GM_* and *M_WM_* were binarised by keeping voxels with probabilities over 0.5; the resulting binary masks were dilated with a 2D 1-voxel structuring element (4-connectivity), and the intersection between the two dilated masks was considered as a mask of the interface between GM and WM. The λ parameter was set as the mean of the gradient magnitude of *mFLAIR* on this “interface” mask.

Non linear diffusion process steady state is never reached in practice. In order to ensure robustness of the algorithm, an interleaved procedure was defined: series of 100 iterations with a time-step of 0.1 were alternated with a watershed segmentation step. The algorithm stopped when two consecutive watershed results were strictly identical. Each area of the final watershed image was labelled with its mean intensity computed on *mFLAIR* (Figure 2.c). Adjacent regions (4-connectivity of border voxels) which mean intensity difference was lower than λ were merged together. This resulted in a piecewise constant image composed of areas separated by at least λ.

#### Selection of segmented regions

In order to select WMH as hyperintense areas compared to normal GM and WM intensity, the intensity threshold needs to be robust with respect to lesion load. In fact, in case of large lesions, estimating the normal GM and WM intensity from *M_GM_* and *M_WM_* may lead to overestimate this value, as spm segmentation will be more likely to include WMH in WM and GM maps. The *mFLAIR* intensity histogram may allow a more robust estimate, as illustrated in Figure S1. This histogram is made of two main modes: one mode for background and CSF, and one mode for “normal GM and WM”. The “normal GM and WM” mode was computed as the second maximum of the histogram, leading to an average intensity value i˜. Hyperintense regions of the piecewise constant image were then selected as those above a threshold set to *T_WMH_* = i˜+2λ.

However, regions thus selected also embedded parts of the cranium, the putamen, or some parts of the cortex that were bright due to partial volume effects or field inhomogeneities. Regions' location with respect to WM thus appeared as critical information to select the regions corresponding to WMH. Tissue maps *M_GM_*, *M_WM_* and *M_CSF_* were binarised by keeping voxels with probabilities over 0.5 and the largest 6-connected component only was used to obtain binary masks. Here the operations were applied in 3D in order to correctly retrieve cortical convolutions as a single connected component. However WMH have the same intensity as GM on T1 images and were often classified in GM or even as CSF at the border of the ventricles; the masks were thus often incorrect around WMH. In order to obtain a WM map more accurate for each subject, voxels classified both in GM or CSF were reconsidered for classification, and hyperintense outliers on *mFLAIR* were added to the WM map. More precisely, as GM intensities are close to WM ones, voxels in the binarised *M_GM_* were considered as hyperintense outliers if their *mFLAIR* intensity was in the highest 5% on the binarised *M_GM_*. For CSF, as its intensity is nulled out on FLAIR images, voxels which *mFLAIR* intensity was higher than the mean intensity on the binarised *M_GM_* were considered as hyperintense outliers. These thresholds were defined empirically. A morphological dilation (1-voxel structuring element, 4-connectivity) was then applied to *M_WM_* conditionally to the outliers, resulting in the corrected WM mask, called *M_WMcorrected_*. As WMH should belong to WM, areas were selected as WMH if more than 50% of their volume was located in *M_WMcorrected_*.

**Figure 3:**
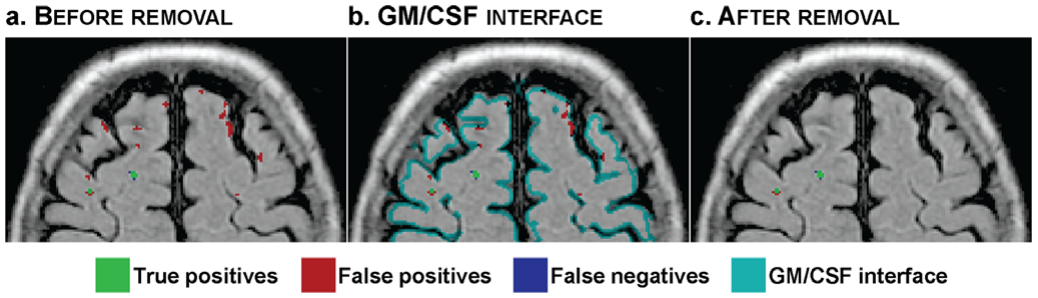
Removal of false positives.

Small false positive hyperintense areas could remain detected as WMH within the cortical ribbon due to its highly convoluted shape. These artefactual hyperintensities could be discriminated based on their location with respect to the GM/CSF interface. Indeed, because of the thinness of the cortex, these areas were close to the GM/CSF interface while true WMH lay within WM, further away from this interface. To remove only the spurious hyperintense areas, the GM/CSF interface was defined as described earlier for GM/WM: *M_CSF_* and *M_GM_* were binarised by keeping voxels with probabilities over 0.5; the resulting masks were dilated by a 2D 1-voxel structuring element (4-connectivity) and the intersection between the two dilated binary regions was computed. Regions that were previously classified as WMH were removed if they were smaller than a given size (*S_FPmax_* = 20 voxels, empirically chosen as large enough for the very limited areas following the cortical ribbon and small enough compared to large lesions extending close to the cortex or near the ventricles in case the WM at the border of the ventricles should be wrongly segmented by spm and appear as GM/CSF interface) and in the same time connected to this GM/CSF interface (4-connectivity) (Figure 3). Despite the bias correction, the brainstem often appears more hyperintense than the other tissues and may thus be classified as WMH. Rather than discarding all WMH from this area, which may result in discarding true WMH, only areas labelled as WMH larger than a given empirical size, *S_Brainstem_* = 50 voxels and intersecting *msP* in the lower slices were removed, as too large to correspond to real lesions.

### Evaluation and comparison study

WHASA was first evaluated by comparing its result with volume and overlap measures with respect to reference segmentations on a multicentre dataset. In order to compare its performances versus state-of-the-art methods, previously published method algorithms were reimplemented and evaluated on the same dataset, which allows a better comparison than using performances estimated on different datasets.

#### Material

Two different datasets were used and combined to cover a wide range of WMH lesion loads and to evaluate robustness with respect to MRI scanner and acquisition settings. Dataset 1: 24 patients with amnestic Mild Cognitive Impairment (MCI) underwent MRI acquisition in five different centres. MRI datasets were acquired within the multicentre *Hippocampus* study [31]. Patients considered here were selected from all the pre-screened subjects in order to cover a wide range of pathological variability. For subsequent comparison with automatic segmentations, manual segmentations of WMH were delineated using the Anatomist visualization module (http://brainvisa.info) and checked by a neuroradiologist. Dataset 2: 43 patients with a monogenic small vessel disease of the brain, CADASIL, were randomly selected from a large cohort of more than 200 patients, from the Lariboisière hospital (Paris). CADASIL is characterized by a large inter-subject variability of WMH lesion load. For all patients, diagnosis was confirmed by the identification of a typical Notch3 mutation. For subsequent comparison with automatic segmentations, lesion maps were generated from FLAIR images using tools provided by BioClinica SAS. After applying an intra-cranial cavity mask, a threshold on signal intensity derived from the histogram was applied. Two trained neuroradiologists then validated each binary lesion and corrected its borders when necessary, using manual editing tools. MR parameters are summarized for both datasets in Table 1.

**Table 1:** MR parameters.

		Machine	Sequence	TR	TE	TI	Flip angle	In-plane resolution (mm)	Slice thickness (mm)
**Dataset 1**	**Centre 1 (N = 6)**	Philips 1.5T	3DT1	7.9	3.7	-	8	1×1	1.3
			FLAIR	10000	140	2200	-	0.94×0.94	5.5
	**Centre 2 (N = 1)**	Philips 1.5T	3DT1	7.9	3.7	-	8	1×1	1.3
			FLAIR	10000	140	2200	-	0.94×0.94	5.5
	**Centre 3 (N = 7)**	Philips 1.5T	3DT1	7.8	3.7	-	8	1×1	1.3
			FLAIR	11000	140	2200	-	0.9×0.9	6.2
	**Centre 4 (N = 3)**	Siemens 1.5T	3DT1	2160	4.3	-	15	1×1	1.3
			FLAIR	8280	111	2500	-	0.9×0.9	4
	**Centre 5 (N = 7)**	General Electric 1.5T	3DT1	11.2	3.5	-	10	0.94×0.94	1.3
			FLAIR	10002	145	2200	-	0.94×0.94	5.5
**Dataset 2**	**Centre 1 (N = 43)**	General Electric 1.5T	3DT1	9	2	-	20	1.02×1.02	0.8
			FLAIR	8402	161	2002	-	0.94×0.94	5.5

#### Evaluation indices & statistical analyses

Unless otherwise stated, all statistical analyses were done with Matlab.

Volume agreement of the automated segmentation (Seg) with reference segmentation (Ref) was evaluated through two-way mixed single measures Intraclass Correlation Coefficient (ICC) given by *Matlab Central* (www.mathworks.com/matlabcentral/fileexchange/21501-intraclass-correlation-coefficients), regression analyses, and Bland and Altman plots.

Bland and Altman plots [32] enabled to visualise consistency between manual and automated volumes by plotting the difference between two measurements, called *D*, versus their average, called *A*. Statistical analyses may then be conducted as follows: the mean difference 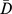 is an estimate of the bias between the two methods and the standard deviation of differences (SD) allows computing limits of agreement between the two methods. Under the assumption that the differences are normally distributed, 95% limits of agreement can be computed as 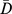
±1.96 SD. However, these estimates are accurate only if bias and variability are uniform throughout the measurement range. In our case, we can assume that measurement error would probably increase with total lesion load. We followed the method proposed in [33] for such cases and tested if the linear relation between *D* and *A* was statistically significant (p-value≤0.05). If so, linear regression gave us 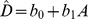. The residuals of this regression, noted *R*, were then used to estimate the variation of the scattering of *D* with respect to lesion load. Absolute values of *R* were regressed on *A*. If the linear relation between *R* and *A* was statistically significant (p≤0.05), one can then write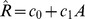 and 95% limits of agreement values were computed as 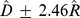. If the relation between *R* and *A* was not significant, 95% limits of agreements were obtained by 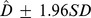.

Spatial agreement was evaluated thanks to the Similarity Index [34]:
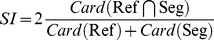



In the comparison study, one-tailed t-tests were performed to evaluate if mean SI values of the implemented methods were statistically different with respect to the mean SI value given by WHASA.

#### Methods selected for comparison & implementation

A thorough comparison to other state-of-the-art methods was undertaken on the same dataset, by reimplementing the methods with the information available in the literature while uniformising the preprocessing steps in order to compare only the algorithms. Two unsupervised and two supervised methods were selected for comparison, based on the work by Klöppel et al [26]. kNN and SVM approaches were evaluated, as they were reported to give better results. Otsu's thresholding was reported to fail dramatically in [26]; we thus adapted instead another thresholding method presented in [18]. Freesurfer software was reported in recent conference paper [35] to be useful for WMH analysis. It is freely available and automatic, and was also included in our comparison study.

#### Additional preprocessing steps

Preprocessing steps described in section 1.1 were applied to all the methods for uniformisation issues. Two more steps were also applied, that were required for some of the reimplemented methods:

- Step 4: A brainmask was created by summing and thresholding as follow: (*M_GM_*+*M_WM_*+*M_CSF_*)≥0.5. 2D morphological opening and closing with a disk shaped structuring element (radius of 2 voxels) was applied to remove isolated groups of voxels and fill small holes.
- Step 5 (for supervised methods): *mT1^FLAIR space^* and *mFLAIR* intensities within the brainmask were normalized to a median of zero and an interquartile range of 1.

#### Unsupervised methods

Freesurfer (http://surfer.nmr.mgh.harvard.edu/) is a set of automated tools for reconstruction of the brain's cortical surface from structural MRI data [36] [37] [38] [39] [40]. Briefly, this processing includes motion correction, removal of non-brain tissue using a hybrid watershed/surface deformation procedure, automated Talairach transformation, segmentation of the subcortical white matter and deep grey matter volumetric structures, intensity normalization, tessellation of the grey matter/white matter boundary, automated topology correction, and surface deformation following intensity gradients to optimally segment borders at the location where the greatest shift in intensity defines the transition to the other tissue class . In its subcortical segmentation procedure, Freesurfer includes a label for WMH. T1 images were processed with Freesurfer and WMH segmentations were obtained in T1 space. They were then registered to FLAIR space using the transformation computed in the preprocessing step 2, in order to compare the results with the references.

Different methods relying on intensity thresholding have been proposed previously for segmenting WMH [17] [18] [41]. We implemented here the method described in [18], which was described with more details than the others. Briefly, the means μ^FLAIR^, μ^T1^ and standard deviations σ^FLAIR^, σ^T1^ of GM, WM and CSF were estimated for *mT1^FLAIR space^* and *mFLAIR*. The WM MNI probability map was then used as a function to weigh FLAIR intensities **FLAIR** *_weighted_* = c×*mFLAIR*×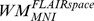, where c is a constant. Hyperintense voxels were detected in **FLAIR** *_weighted_* if their intensities satisfied the following criterion: 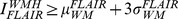. Remaining false positives were removed by examining the corresponding T1 image: 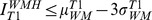. The setting of the c constant was not described in [18]; the only indication is that it is a constant greater than 2. Thus, in order to find the best value, we let it vary from 1.5 to 3.5 with a step of 0.1, and computed the evaluation indices for each value for all the subjects. The best constant c was chosen as the one which gave the best mean SI on the whole dataset.

#### Supervised methods

The choice of the learning set is a crucial step for supervised methods since it should represent the whole range of variability; it may thus be difficult to select a representative dataset that allows generalisation to new images. Given the variability of our two datasets, the performance of the two algorithms was evaluated with three different learning sets.

- Learning Set 1: 10 patients chosen among the *Hippocampus* dataset to sample the variability between centres and lesion load.
- Learning Set 2: 10 patients chosen among the CADASIL dataset to sample the variability of lesion load.
- Learning Set 3: five patients chosen among the *Hippocampus* dataset and five patients randomly chosen among the CADASIL dataset to sample the variability between centres and lesion load.

Limits on computation time and memory load prevent from selecting all voxels for all subjects in the learning set. The procedure to choose relevant voxels for training was derived from [26]. 500 WMH voxels were randomly selected for each patient. For non-WMH voxels, the boundary at a 5 mm distance from WMH was computed. 250 voxels were randomly chosen inside this boundary and 250 outside. The total number of training voxels for each learning set, composed of ten patients, was thus 10.000.

The feature vector should contain information considered as relevant for the classification problem. For WMH segmentation, relevant data may be divided into two categories: intensity and spatial information. While Anbeek et al [23] used only the intensity in the voxel candidate for classification, the neighbouring voxels were included either as belonging to a cube [25] or to a sphere [26]. More precisely, Klöppel et al [26] defined the neighbourhood as an 8 mm radius sphere discretized for a 1×1×6.25 mm3 voxel; FLAIR images were previously interpolated from 0.5×0.5×6.25 mm3 voxels to this voxel size.

In our datasets, images have different slice thicknesses. We defined a reference voxel size, 0.9375×0.9375×5.5 mm^3^, since it was the median voxel size across the different protocols. In order to take into account adjacent superior and inferior slices, the neighbourhood was defined as the discretization of a sphere of 8 mm radius into this reference grid, resulting in 466 neighbouring voxels (467 when including the central voxel) (See Figure S2). In order to ensure a consistent feature vector length across the datasets, this neighbourhood pattern was then used on all images, regardless their actual voxel size.

In order to embed coherent spatial information, corresponding MNI coordinates of the voxel were computed using affine transform as in [26]. Each coordinate was scaled between −1 and 1 by dividing by the MNI space dimensions for ensuring a similar range with respect to intensity features.

Four different feature vectors (FV) were evaluated.

- FV A: 3-dimension vector composed of FLAIR intensity, T1 intensity and *WM_MNI_* probability.
- FV B: 6-dimension vector composed of FLAIR intensity, T1 intensity, *WM_MNI_* probability and spatial coordinates in Talairach space.
- FV C: 1401-dimension vector composed of FLAIR intensity of the voxel and its neighbourhood, T1 intensity of the voxel and its neighbourhood and *WM_MNI_* probability of the voxel and its neighbourhood.
- FV D: 1404-dimension vector composed of FLAIR intensity of the voxel and its neighbourhood, T1 intensity of the voxel and its neighbourhood, *WM_MNI_* probability of the voxel and its neighbourhood and spatial coordinates in Talairach space.

To make both intensity and spatial information equally important, each scaled MNI coordinate was normalised with respect to the number of features extracted from imaging modalities, as explained in Appendix S1.

KNN classification for WMH segmentation was proposed in [23]. In this approach, a new voxel is classified depending on the labels of its k closest neighbours in the feature space using Euclidian distance. Anbeek et al [23] proposed to set k = 100 as the best compromise between computation time and accuracy. Rather than a simple majority vote, the result of the kNN in each voxel is the proportion of voxels classified as WMH in the k nearest neighbours, which can be interpreted as a probability for each voxel to be classified as WMH. In our implementation, the optimal threshold for the probability map is obtained by finding the highest mean SI on the images used to build the learning set.

SVM is a powerful tool for high dimensional classification [42]; its use is increasing steadily in neuroimaging. Application of SVM to the problem of WMH segmentation has been proposed in [25] [43] [26]. We used the freely available LIBSVM library (http://www.csie.ntu.edu.tw/~cjlin/libsvm/). Given the low dimension of the feature space, classification was performed using the C-SVM implementation with Radial Basis Functions (RBF) kernels: 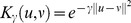, where the *γ* parameter *c*ontrols the width of the kernel. This kernel was also used in [26] and [25]. Hyperparameters *γ* and *C* were optimized by a 2-fold cross-validation grid-search on the learning set. A first coarse grid was constructed on a wide range (from −5 to 13 with a step of 2 for log_2_(*C)* and from −21 to 5 with a step of 2 for log_2_(*γ))*. The best (*C_max_*,*γ_max_*) couple was identified as the one with the best cross-validation accuracy. A finer grid search was then conducted on the subset ([2*^Cmax^* ^−2^, 2*^Cmax^* ^+2^], [2*^γmax^* ^−2^, 2*^γmax^* ^+2^]) with a 0.25 step on a logarithmic scale. The final best couple was obtained as the one with the best cross-validation rate on this finer grid. The final classifier was generated using this best couple on the whole learning set. The classification step results in an image in which the value of the classification function (or decision value) is computed at each voxel. In order to obtain the binary segmentation, a cut-off needs to be defined for this decision value; the usual value of the cut-off is zero, since the hyperplan given by the SVM is optimal for the voxels included in the learning set. However, in our case, the aim is not to obtain the optimal hyperplane for 1000 voxels in each image but the optimal final segmentation of each image; the hyperplane as obtained by the SVM with this learning set may thus be suboptimal for the segmentation problem. Klöppel et al [26] suggested thus optimizing the cut off of the decision value with respect to the measure of interest. Similarly to the threshold of the kNN result, the optimal cut-off was thus defined as the one with the best mean SI on the images used to build the learning set. Since the hyperplane was supposed to be a good estimate of the optimal segmentation, the cut-off was varied between −5 and 5 with a 0.25-step.

## Results

WHASA was first qualitatively and quantitatively evaluated on the two datasets described above. The same analysis was then undertaken for the reimplemented methods. Finally, all the results were compared with WHASA's.

### WHASA results

**Figure 4:**
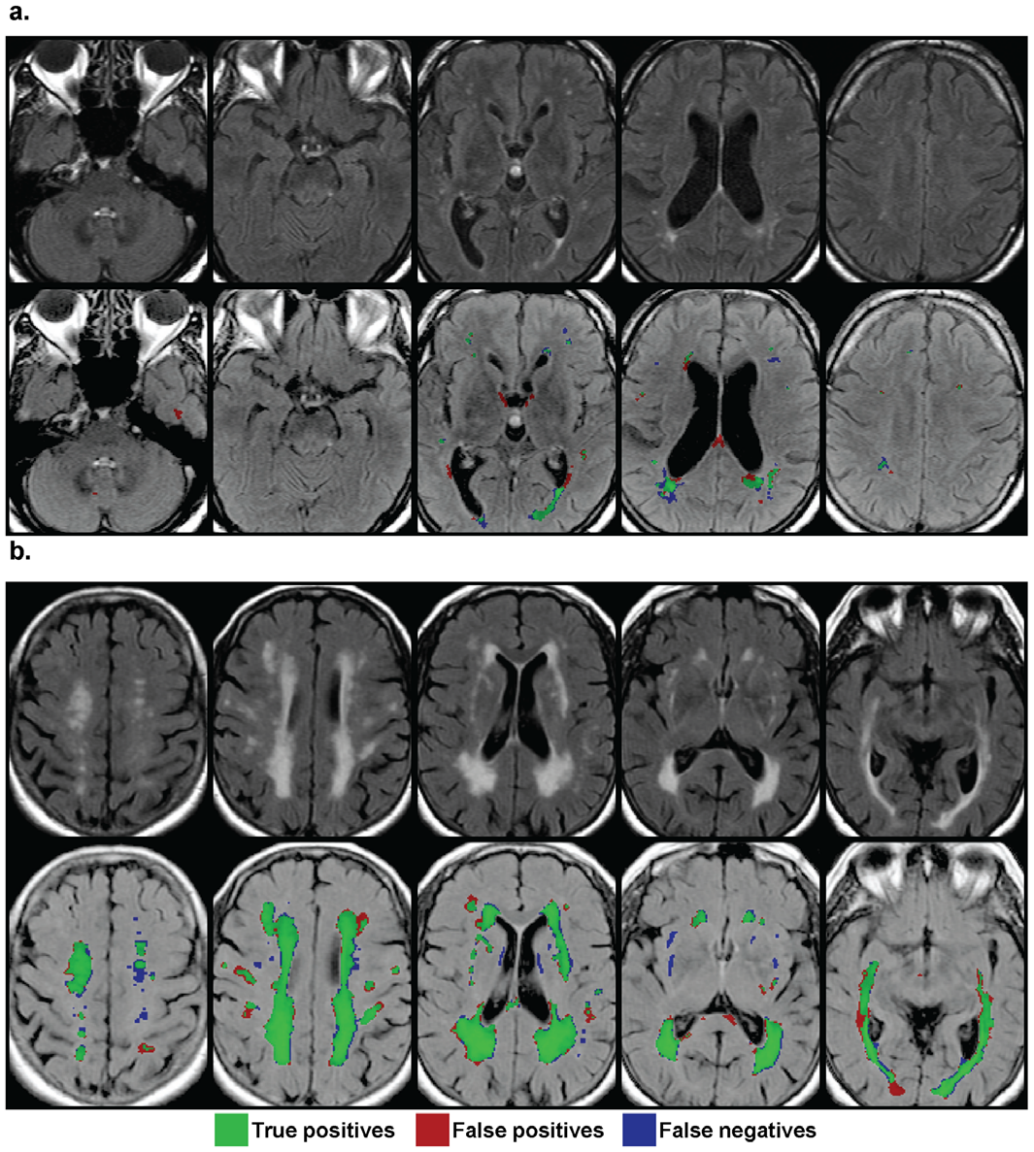
Illustration of WHASA results. In order to obtain representative results, the subjects with the highest and the lowest SI amongst subjects with a total lesion load between 10 and 80 cm^3^ are displayed. **a.** Subject with lowest performance. (Reference volume: 10.5 mL; WHASA volume: 11.7 mL; SI = 0.52). **b.** Subject with highest performance (Reference volume: 72.6 mL; WHASA volume: 73.8 mL; SI = 0.85).

WHASA results were visually checked and showed coherent behaviour, as illustrated in Figure 4.

**Figure 5:**
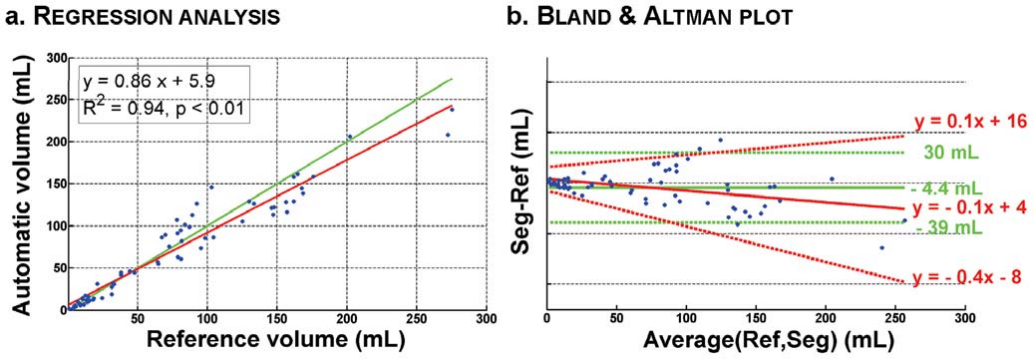
WHASA results for quantitative evaluation. **a.** Regression line is shown in red, identity line in green. **b.** Mean bias and 95% limits of agreement computed for uniform variability are shown in green. Mean bias and 95% limits of agreement when taking into account differences variability with respect to magnitude are shown in red (For more details, see text in section “Evaluation indices & statistical analyses”).

Volume agreement evaluation led to an ICC value of 0.96, indicating good performance for volume analysis. Linear regression analysis, shown in Figure 5.a, resulted in a regression coefficient value of R = 0.97 and a regression slope of 0.86. Bland and Altman plot (Figure 5.b) showed a slight underestimation (bias of −4.4 mL) and a 95% interval of [−39 mL 30 mL]. This interval is only a rough estimate of the agreement: Figure 5.b. clearly reveals the influence of lesion load on the agreement between WHASA and reference volumes. Indeed, the mean bias is slightly positive for small lesion load and decreases slowly when lesion load increases; 95% limits of agreement are quite narrow for small lesion load and widen when lesion load increase. This could be expected, as the number of uncertain voxels, even for manual segmentation, increases as the lesion volume and surface increase.

Spatial analysis led to a mean SI value and standard deviation for the whole evaluation set of 0.72±0.16.

### Implemented methods

The methods that were implemented for the comparison study were first evaluated as WHASA. Results for all the methods with all the sub-cases are summarized in Table 2.

**Table 2:** Quantitative evaluation of all methods on different test sets.

				Regression analysis			Bland and Altman	
	Method	Mean SI±SD	ICC	Slope	y-intercept	R^2^	Bias	95% limits
**Fulldataset** **(unsupervisedmethods)**	WHASA	0.72±0.16	0.96	0.86	5.9	0.94	−4.4	[−39 30]
	FS	0.40±0.13	0.52	0.29	4.64	0.82	−49	[−144 47]
	Threshold	0.54±0.15	0.53	0.29	12.16	0.80	−41	[−136 54]
**Test Set 1**	WHASA	0.74±0.15	0.96	0.85	8.3	0.93	−4.5	[−42 33]
	Best kNN	0.71±0.19	0.90	0.79	29	0.83	11	[−44 66]
	Best SVM	0.72±0.16	0.89	0.68	17	0.88	−10	[−65 44]
**Test Set 2**	WHASA	0.71±0.16	0.96	0.87	5.3	0.93	−3.2	[−36 30]
	Best kNN	0.63±0.22	0.87	0.73	19	0.79	0.3	[−56 57]
	Best SVM	0.67±0.18	0.92	0.78	9.4	0.87	−5.1	[−51 41]
**Test Set 3**	WHASA	0.71±0.17	0.96	0.86	6.5	0.93	−4.2	[−41 32]
	Best kNN	0.70±0.19	0.91	0.75	22	0.86	3.0	[−49 55]
	Best SVM	0.70±0.19	0.94	0.80	15	0.90	−0.9	[−45 43]

#### Unsupervised methods

Visual evaluation for Freesurfer and the thresholding approach showed poor delineation of WMH. (See File S1). Regarding volume agreement evaluation, ICC values were respectively 0.52 and 0.53 for Freesurfer and the thresholding approach. The regression analysis resulted in values of 0.29 for the slope of the regression line in both cases (See File S1). Spatial agreement evaluation yielded mean SI values of 0.40 and 0.54.

These results indicated very poor performance, for both volume and spatial agreement. Bland and Altman plots were thus not shown for these two methods.

#### Supervised methods

In order to proceed to an unbiased evaluation of supervised methods, we took into account in the evaluation only subjects that were not included in the learning sets. Since we had three different learning sets, each of them made of 10 patients; evaluation was performed on the 57 remaining patients, referred to as test sets.

For each test set, we first compared the different possibilities for constructing the feature vector (See File S2). Over the three test sets, kNN performed lower with feature vectors C and D (longer feature vectors that included neighborhood information). Better results were obtained for kNN with feature vectors A and B (no neighborhood information), both giving very similar results. SVM performance was stable with feature vectors A, B and C but dropped with feature vector D (neighborhood and spatial coordinates) for test sets 2 and 3.

**Figure 6:**
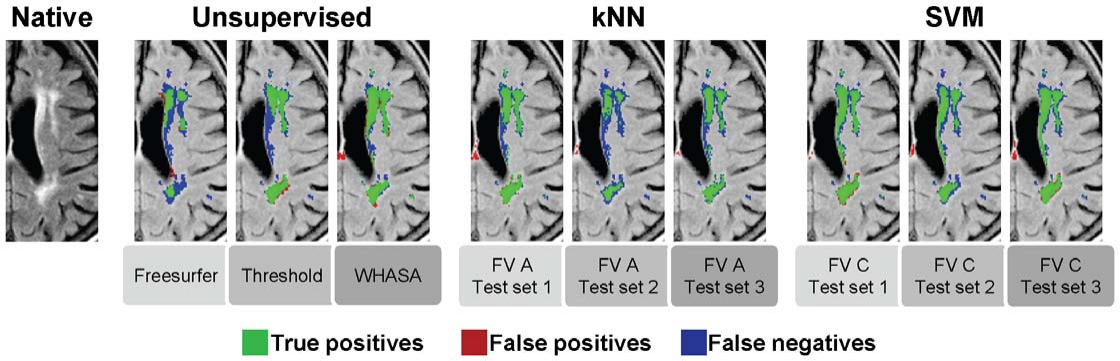
Representative slice showing segmentation results for all methods. Reference volume: 31.5 mL; Freesurfer (Volume = 10.5 mL, SI = 0.40) ; Thresholding (Vol = 17.9 mL, SI = 0.70) ; WHASA(Vol = 26.6 mL, SI = 0.79) ; kNN test set 1 (Volume = 24.6 mL, SI = 0.81) ; kNN test set 2 (Volume = 10.7 mL, SI = 0.48) ; kNN test set 3 (Volume = 18.7 mL, SI = 0.71) ; SVM test set 1 (Volume = 26.3 mL , SI = 0.82) ; SVM test set 2 (Volume = 16.7 mL , SI = 0.67) ; SVM test set 3 (Volume = 25.1 mL , SI = 0.80).

Since the behavior was very similar between different feature vectors, we focused on one of them for each test set for further analysis. kNN best results were obtained with feature vector A (highest mean SI for the three test sets) and SVM best results were obtained with feature vector C (highest mean SI and ICC for test set 1 and 2, highest ICC and second highest mean SI for test set 3). Segmentations obtained with these feature vectors for kNN and SVM are shown in Figure 6 and appeared dependent on the test set used. This effect was particularly visible for kNN.

**Figure 7:**
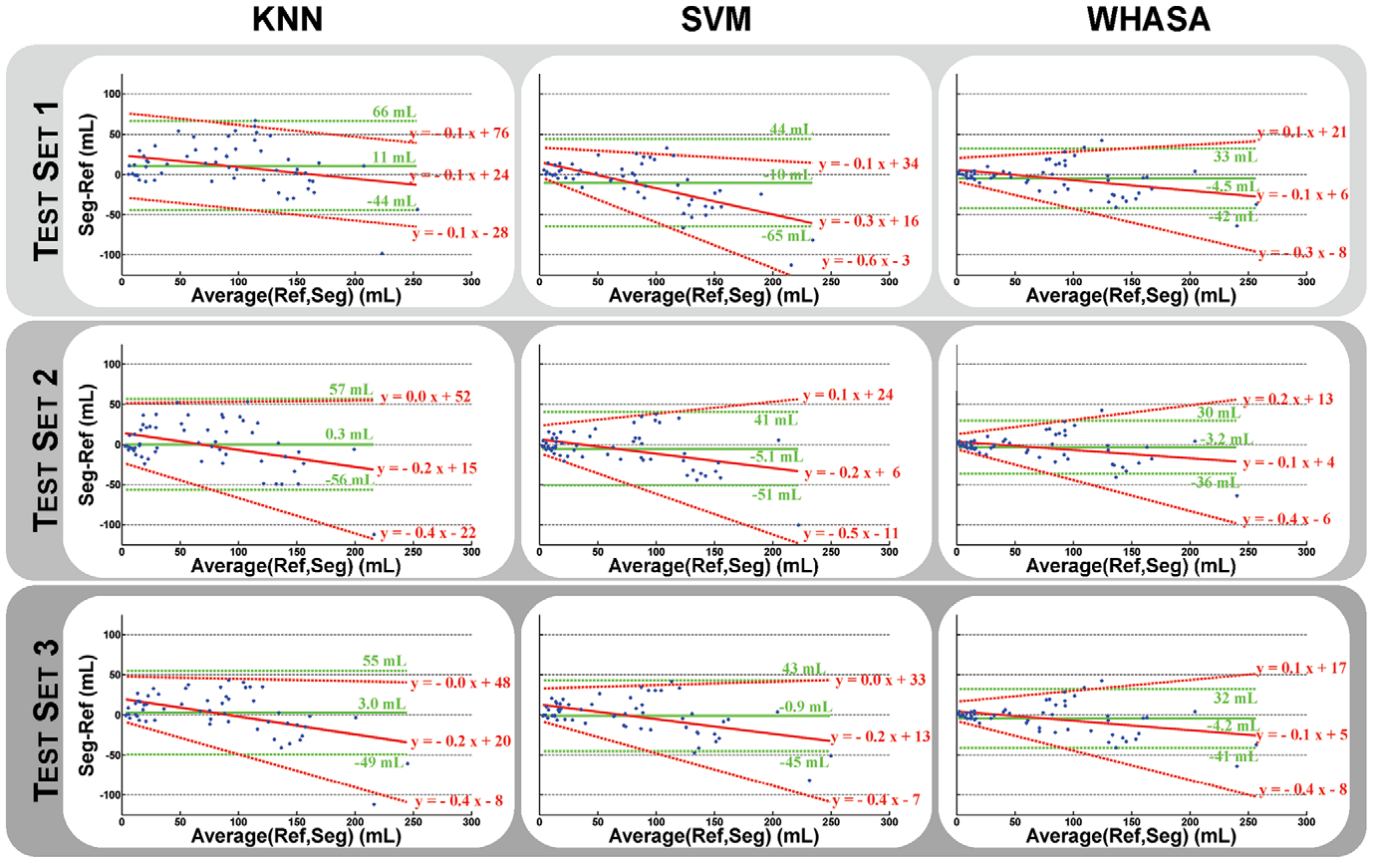
Bland and Altman plots comparing supervised methods and WHASA for different training sets/test sets. Mean bias and 95% limits of agreement computed for uniform variability are shown in green. Mean bias and 95% limits of agreement when taking into account differences variability with respect to magnitude are shown in red (For more details, see text in section “Evaluation indices & statistical analyses”).

ICC values were 0.90 for test set 1, 0.87 for test set 2 and 0.91 for test set 3 for kNN, and respectively 0.89, 0.92 and 0.94 for SVM. Bland and Altman plots for kNN and SVM for each training set/test set are shown in Figure 7. kNN tends to always overestimate lesions (mean bias ranging between 0.3 and 11 mL), while SVM tends to underestimate them in all cases (mean bias range: −10 to −0.9 mL). When taking into account relationship between measurement and lesion load, the mean bias decreased in all cases. 95% limits of agreement were quite broad even for small lesions for kNN. SVM was more influenced by the lesion load for test set 1 while behaving similarly for test set 2 and 3.

Mean SI ± SD were respectively 0.71±0.19, 0.63±0.22 and 0.70±0.19 for kNN and 0.72±0.16, 0.67±0.18 and 0.70±0.19 for SVM. One can notice a lower performance for training set 2, which is composed of images from CADASIL patients only, that is statistically significant for kNN only.

### Methods comparison

Table 3 shows the computational time for each method. They varied widely from a few seconds for thresholding up to five hours for SVM with high dimensional feature vectors. WHASA belongs to the fastest methods, being slower than threshold and kNN with short feature vectors. For supervised methods, training time is most of the time limited to few minutes but may take up to 20 hours for SVM because of the search grid for optimizing parameters. Note that training has to be performed only once.

**Table 3:** Computational time of the different methods.

			Training time	Testing time (per subject)
**Unsupervised**	**Freesurfer**		None	∼24 hours
	**Threshold**		None	<1 minute
	**WHASA**		None	∼30 minutes
**Supervised**	**kNN**	Feature Vector A/B	Minutes	∼10 minutes
		Feature Vector C/D	Minutes	20–30 minutes
	**SVM**	Feature Vector A/B	Minutes	1–2 minutes
		Feature Vector C/D	15–20 hours	3–5 hours

**Figure 8:**
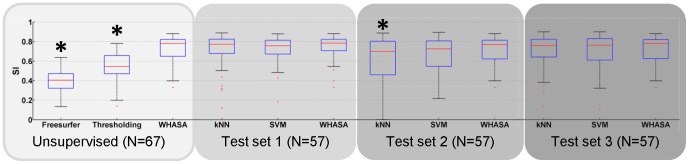
SI distribution for all methods. *: significantly lower than WHASA methods (one-tailed t-test, p<0.05).

As shown by the results, WHASA outperformed the two other unsupervised methods in the comparison study. Mean SI were statistically higher for WHASA than for Freesurfer (32 percentage points) or the thresholding approach (18 percentage points) as tested with one-tailed t-test, as displayed in Figure 8.

In order to compare methods on the same test set used for supervised methods, the evaluation was also restricted to the test sets for WHASA, as shown in Figure 7 and Figure 8. Bland and Altman plots allow refining the analysis of the differences between methods. The average error was more stable between training set/test set for WHASA. Furthermore, 95% limits of agreement, when taking into account variability with respect to lesion load, were always narrower for WHASA. Mean SI were less stable between training sets/test sets and slightly lower for the supervised methods (between 8 and 1 percentage points for the kNN and 4 and 1 percentage points for the SVM), although the difference was only statistically significant for the kNN approach with training set/test set 2.

## Discussion

We have presented WHASA, a new method for automatically segmenting white matter hyperintensities from FLAIR and T1 images in multi centre studies. This method relies on contrast, emphasized through non linear diffusion filtering, and robust anatomical prior information, using a combination of morphological and segmentation steps. It has been evaluated on 67 patients from two studies, acquired on six different MRI scanners and displaying a wide range of lesion load, with volume and spatial agreement measures with respect to reference segmentation. Its performances have been compared with four methods, with considerably better results with respect to two other unsupervised methods, and similar or better results compared with two optimised supervised approaches.

### WHASA evaluation, parameter setting, strengths and limits

One of the main strengths of WHASA for multicentre studies is the very small number of parameters involved in the algorithm. This results from a contrast-based approach that is closer to the specificity of visual detection of WMH than intensity-based methods. The contrast characteristics rely on the contrast parameter λ, which is automatically computed for each subject. This allows a contrast improvement preliminary step through non linear diffusion filtering; the detection of hyperintensities then becomes much less sensitive to the intensity threshold *T_WMH_*. Improvements could still be considered, for example through a better estimation of λ on a slice-by-slice basis, which would make it less sensitive to 2D acquisition issues.

The definition of outliers to correct the white matter mask relies on stronger assumptions which make parameter setting more difficult. Whereas hyperintense outliers in CSF are obviously detected using the FLAIR image, the continuum between GM and WM intensities on FLAIR images yields a more difficult process for outliers in GM. The intensity parameter was set to the highest 5% of intensities of M_GM_, this definition being commonly used to characterize outliers in statistics. Two parameters were used for removing specific false positives from the segmentation results: *S_FPmax_* and *S_Brainstem_*. These are used for removing hyperintense voxels in very specific areas (respectively near the cortex and in the brainstem); they were set empirically. Robustness of these parameters was demonstrated by the coherent behaviour of WHASA on patients with a very wide range of lesion load and on different MRI scanners. Indeed, the method was designed on 24 subjects from dataset 1 and empirical parameters were set for these subjects, without being subsequently modified for the other subjects, even though the range of lesion load was markedly different.

Consistent results were obtained with WHASA on this widely varied population, which further illustrates the reliability of the method. Intraclass correlation coefficient, regression analyses and Bland and Altman limits all underlined the consistency of excellent volume agreement, for small and large lesion loads. Mean SI value was in all cases above 0.7, indicating good spatial agreement.

Although WHASA was designed to be robust to parameter acquisitions, we did not have enough data at 3T with reference segmentation to evaluate the performance of the algorithm on such data. Preliminary experiments on some images at 3T suggest that the algorithm is also robust to field strength, provided that bias correction was efficient.

### Comparison with other methods

A large number of methods have been proposed for WMH segmentation; however, different metrics and different datasets were used for the evaluation, which makes fair comparison impossible. Therefore, we conducted a thorough comparison study to compare WHASA with two unsupervised and two supervised previous approaches on the same dataset. The two unsupervised approaches were FreeSurfer and a thresholding method. Freesurfer is widely used in neuroimaging research and embeds an estimate of WMH from T1 images that has been used for WMH analysis in [35]. Different methods have also been proposed to segment WMH mainly by thresholding [17] [18] [41]; the method in [18] was chosen because enough details were provided to allow reimplementing it. kNN and SVM were two supervised methods proposed for WMH segmentation [23] [25] [26], with different optimization strategies and feature vectors' definition. We adapted the framework detailed in [26] to establish an unbiased and meaningful comparison.

Unsupervised methods other than WHASA showed significantly lower performances than supervised ones, which is in accordance with the results presented in [26]. As previously reported in [44], disappointing results were obtained with Freesurfer. Note that Freesurfer primary goal is not WMH segmentation and that it relies only on T1 images, on which WMH are poorly defined. The thresholding method implemented here gives better but still unsatisfactory results. The optimal threshold in [18] is derived from the standard deviation of FLAIR intensities on GM. For large lesion loads, T1 based GM segmentation will be more likely to include a large number of WMH; this will result in an overestimation of the standard deviation of FLAIR intensities on GM, and thus of the threshold. Otsu's thresholding method was evaluated in [26], with similar disappointing results. In fact, it is likely that any segmentation derived from thresholding the original FLAIR image will fail, due to the overlap between tissues and WMH intensities on FLAIR images.

As far as supervised methods are concerned, the first step was to determine the most relevant features. While Anbeek et al [23] only used intensities from five MR sequences and spatial coordinates, Lao et al [25] included neighbourhood information but no spatial coordinate. Klöppel et al [26] only used T1 and FLAIR images together with a WM probability map, but also investigated the benefit of introducing new information derived from Gabor filters. As WHASA only uses T1, FLAIR and probability WM, GM and CSF maps, we decided to use the same input images and maps for supervised methods and also evaluated the influence of neighbourhood information and spatial coordinates. For kNN, including neighbourhood information led to lower results; the feature vector dimension then became large, and kNN is not designed for high dimensional classification. Unlike Anbeek et al [23], no improvement was observed here when introducing spatial coordinates in the feature vector. Note that a white matter probability map was included here in all feature vectors, which was not the case in [23]. It is likely that more information is embedded in WM probability maps compared to mere spatial coordinates. For SVM, introducing both spatial coordinates and neighbourhood information gave significantly lower results. Note that Klöppel et al [26] obtained similar results (SI value of 0.56) using both spatial coordinates and neighbourhood information amongst other features. Using “raw” (x,y,z) spatial coordinates within a composite feature vector may not be optimal and more subtle strategies may be devised to incorporate spatial information into the SVM.

Klöppel et al [26] reported much better results with SVM than kNN. This finding was not confirmed by our study, although SVM seemed to perform slightly better than kNN and to be more robust with respect to training/test set. In fact, three different training sets were considered, in order to evaluate the generalizability of supervised methods. The results appeared sensitive to the training set, which was expected, as WMH size, appearance and location are highly variable. kNN performance significantly decreased when using a training set made of patients with large lesion loads. With the same training set, SVM performance also decreased although it was not statistically significant. SVM appeared thus more stable with respect to training set. This variability may be due to the test set differences, performances being computed on the “remaining” subjects; in fact, relative indices such as SI will show lower performance for lower lesion loads. Nevertheless, WHASA performances were less variable than supervised methods' for the three test sets, even if some variability could be observed. Note that, for SVM, better generalizability may probably be obtained by a better selection of training samples, in order to only train the classifier on the “most difficult” samples. On the other hand, such a strategy would increase computational time which would become prohibitive.

### Limits of the comparison study

One goal of the comparison study was to evaluate robustness of methods to different acquisition parameters and different type of scanners. Although data was gathered from five different centres and from three different manufacturers, the pooled dataset was unbalanced with two third of images that actually came from dataset 2 and were acquired on the same scanner (General Electric). This may have led to biased performance towards GE scanners. Unfortunately the number of patients was not high enough to allow a fair estimation of such bias.

While methods for WMH segmentation have only been recently developed, similar research has been conducted for much longer for Multiple Sclerosis (MS) lesions segmentation. MS lesions characteristics differ from WMH in terms of both contrast and location. In fact, their contrast on FLAIR images is often stronger with WM than for WMH and they may also be found in grey matter. Methods developed for segmenting MS lesions have not been thoroughly evaluated for WMH and may not be reliable when applied for WMH. One of the most successful approaches for MS segmentation, used in a clinical trial, was proposed in [45] and relied on artificial neural network (ANN). This supervised method was adapted in order to segment WMH for the LADIS study [46], but showed low spatial agreement (mean SI ± SD ranging from 0.45±0.15 for total lesion load less than 10 ml to 0.65±0.15 for total lesion load larger than 30 ml). Note that this method has not been used for investigating clinical relationships with WMH in the LADIS study. ANN approach was not used in our study as its implementation depends on a high number of parameters and two other supervised approaches were already evaluated.

Other methods specific for WMH segmentation were proposed in the literature such as [21] and [19]. These were not implemented in our study because details regarding implementation issues and parameter setting were too scarce to allow truthful in-house implementation. Maillard et al [21] did not evaluate their segmentation with respect to a reference, but through indirect validation by correlation analyses with clinical variables of interest. Furthermore, their method did not incorporate FLAIR images. Admiraal-Behloul et al [19] reported a mean SI value of 0.75 on 100 elderly subjects but this evaluation was carried on data collected in a single centre, and one cannot conclude with respect to the generalizability of the method for data acquired on other MRI scanners. One should remain cautious when comparing these results with those presented here, since the methods were evaluated on different datasets.

## Conclusions

WHASA is a new method for automatically segmenting white matter hyperintensities from FLAIR and T1 images in multi centre studies, for which it proved to be reliable and robust. This method relies on contrast, emphasized through non linear diffusion filtering, and robust anatomical prior information, using a combination of morphological and segmentation steps. This contrast-based approach enables the use of WHASA on new data without new parameter setting. Evaluation was carried out on 67 patients from two studies, acquired on six different MRI scanners and displaying a wide range of lesion load, with volume and spatial agreement measures with respect to reference segmentation. Its performances have been compared with four methods, with considerably better results with respect to two other unsupervised methods, and similar or better results compared with two optimised supervised approaches.

## Supporting Information

Appendix S1 **Computation of the weights for spatial coordinates.**
(DOC)

Figure S1 **Robustness of the GM/WM mode on the FLAIR histogram.**
(DOC)

Figure S2 **Neighbourhood pattern obtained as the discretization of an 8 mm radius sphere into a reference grid of 0.9375×0.9375×5.5 mm^3^.**
(DOC)

File S1 **Detailed results for Freesurfer and Thresholding methods.**
(DOC)

File S2 **Results** **of supervised methods for all feature vectors and all test/training sets.**
(DOC)
