# Supplementary material for: Contrast-Based Fully Automatic Segmentation of White Matter Hyperintensities: Method and Validation
Source: PLoS One. 2012 Nov 12;7(11):e48953. doi: 10.1371/journal.pone.0048953 (PMC3495958; doi:10.1371/journal.pone.0048953)
Supplement: Appendix S1 — Computation of the weights for spatial coordinates. (DOC) [file pone.0048953.s001.doc]

# Appendix S1 - Computation of the weights for spatial coordinates

kNN uses Euclidian distance to compute the k-nearest neighbours and kernel of the SVM.

Given two vectors X and Y of dimension d, the Euclidian distance is written as:

$$\left\|X-Y\right\|^{2}=\sum_{i=1}^{d} \left(x_{i}-y_{i}\right)^{2}$$

Suppose the feature vector is composed of spatial information (3 coordinates) and information from two MR sequences, e.g. T1 and FLAIR, with N the number of voxels extracted from one modality (i.e. the size of the neighbourhood).

$$\sum_{i=1}^{3} \left(x_{i}^{spatial}-y_{i}^{spatial}\right)^{2}+\sum_{i=1}^{N} \left(x_{i}^{T1}-y_{i}^{T1}\right)^{2}+\sum_{i=1}^{N} \left(x_{i}^{FLAIR}-y_{i}^{FLAIR}\right)^{2}$$

Let’s note p_spatial_, p_T1_, p_FLAIR_ the associated weights when scaling the coordinates:

$$\sum_{i=1}^{3} \left(p_{spatial}x_{i}^{spatial}-p^{spatial}y_{i}^{spatial}\right)^{2}+\sum_{i=1}^{N} \left(p_{T1}x_{i}^{T1}-p_{T1}y_{i}^{T1}\right)^{2}+\sum_{i=1}^{N} \left(p_{FLAIR}x_{i}^{FLAIR}-p_{FLAIR}y_{i}^{FLAIR}\right)^{2}$$

This yield to

$$3p_{spatial}^{2}\sum_{i=1}^{3} \left(x_{i}^{spatial}-y_{i}^{spatial}\right)^{2}+Np_{T1}^{2}\sum_{i=1}^{N} \left(x_{i}^{T1}-y_{i}^{T1}\right)^{2}+Np_{FLAIR}^{2}\sum_{i=1}^{N} \left(x_{i}^{FLAIR}-y_{i}^{FLAIR}\right)^{2}$$

If one wants the spatial information to have the same influence as one modality, this gives us:

$$3p_{spatial}^{2}=Np_{T1}^{2}=Np_{FLAIR}^{2}$$

And therefore for $p_{T1}=1$, one gets $p_{spatial}=\sqrt{\frac{N}{3}}$
