# Supplementary material for: Contrast-Based Fully Automatic Segmentation of White Matter Hyperintensities: Method and Validation
Source: PLoS One. 2012 Nov 12;7(11):e48953. doi: 10.1371/journal.pone.0048953 (PMC3495958; doi:10.1371/journal.pone.0048953)
Supplement: Figure S1 — Robustness of the GM/WM mode on the FLAIR histogram. (DOC) [file pone.0048953.s002.doc]

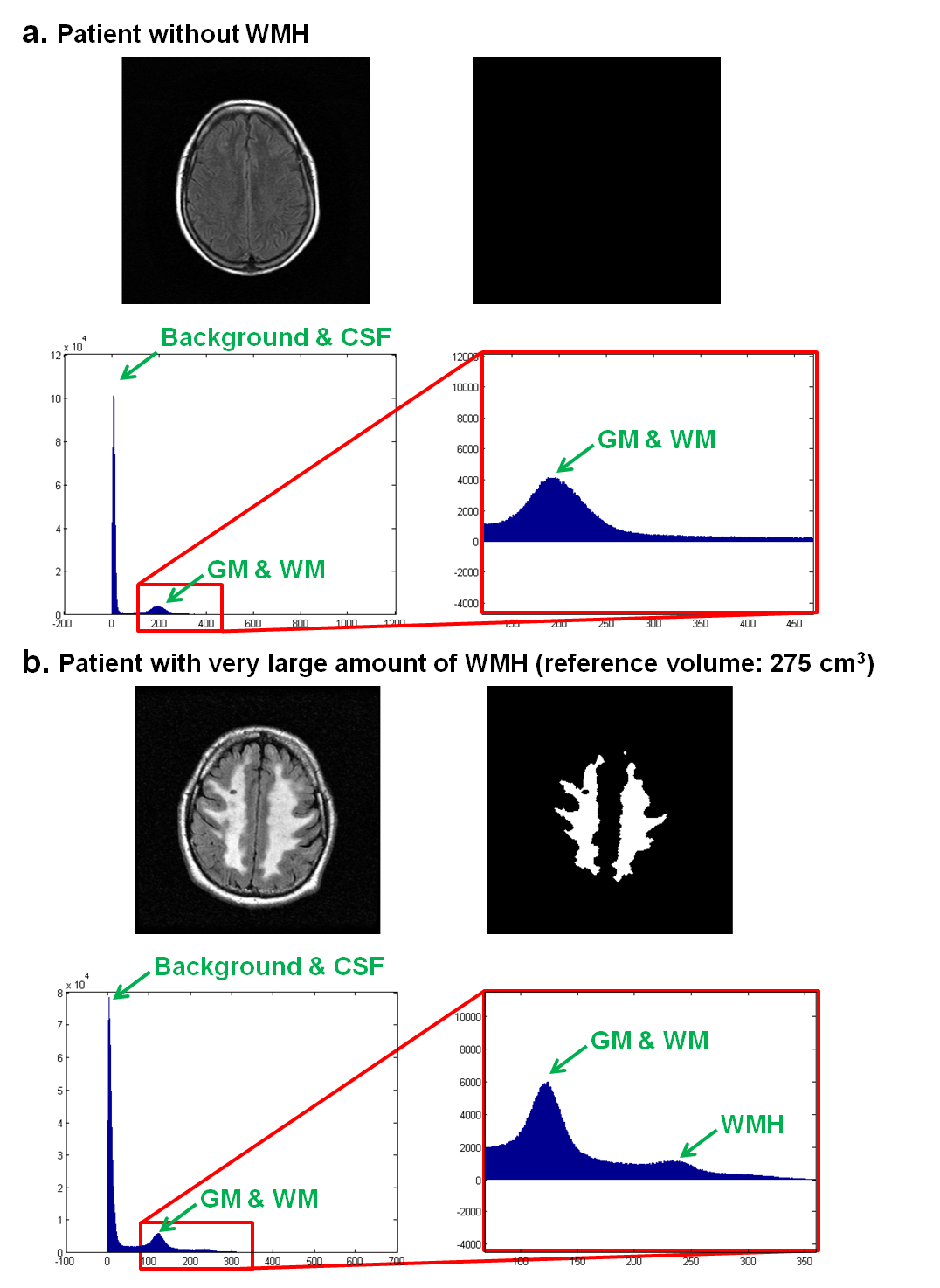


Figure S1 – Robustness of the GM/WM mode on the FLAIR histogram.

The two examples shown here are the two most extreme cases of the database in terms of lesion load. For each patient, it displays one slice of the original FLAIR image (top left), the result of WHASA segmentation (top right), the FLAIR intensity histogram (bottom left) and a zoom on the “normal GM and WM” mode of the histogram (bottom right).
