# Supplementary material for: Contrast-Based Fully Automatic Segmentation of White Matter Hyperintensities: Method and Validation
Source: PLoS One. 2012 Nov 12;7(11):e48953. doi: 10.1371/journal.pone.0048953 (PMC3495958; doi:10.1371/journal.pone.0048953)
Supplement: Figure S2 — Neighbourhood pattern obtained as the discretization of an 8 mm radius sphere into a reference grid of 0.9375×0.9375×5.5 mm3. (DOC) [file pone.0048953.s003.doc]

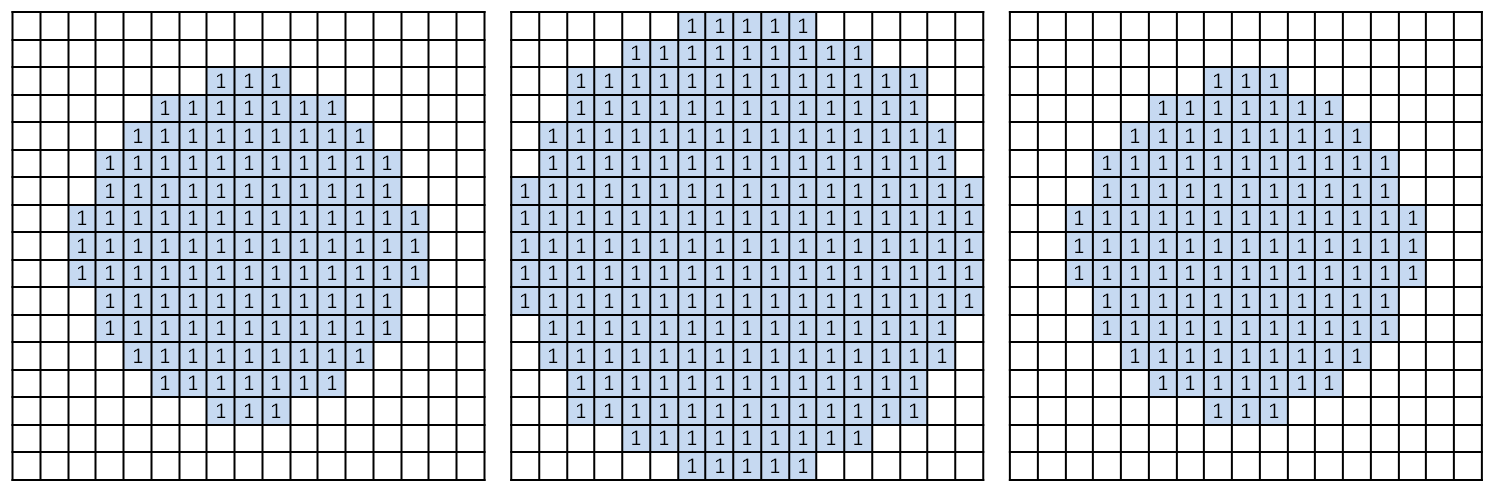


Figure S1 – Neighbourhood pattern obtained as the discretization of an 8mm radius sphere into a reference grid of 0.9375 x 0.9375 x 5.5 mm^3^
