# Supplementary material for: Contrast-Based Fully Automatic Segmentation of White Matter Hyperintensities: Method and Validation
Source: PLoS One. 2012 Nov 12;7(11):e48953. doi: 10.1371/journal.pone.0048953 (PMC3495958; doi:10.1371/journal.pone.0048953)
Supplement: File S1 — Detailed results for Freesurfer and Thresholding methods. (DOC) [file pone.0048953.s004.doc]

**Supporting information File S1:**

**Detailed results for Freesurfer and Thresholding methods.**

**
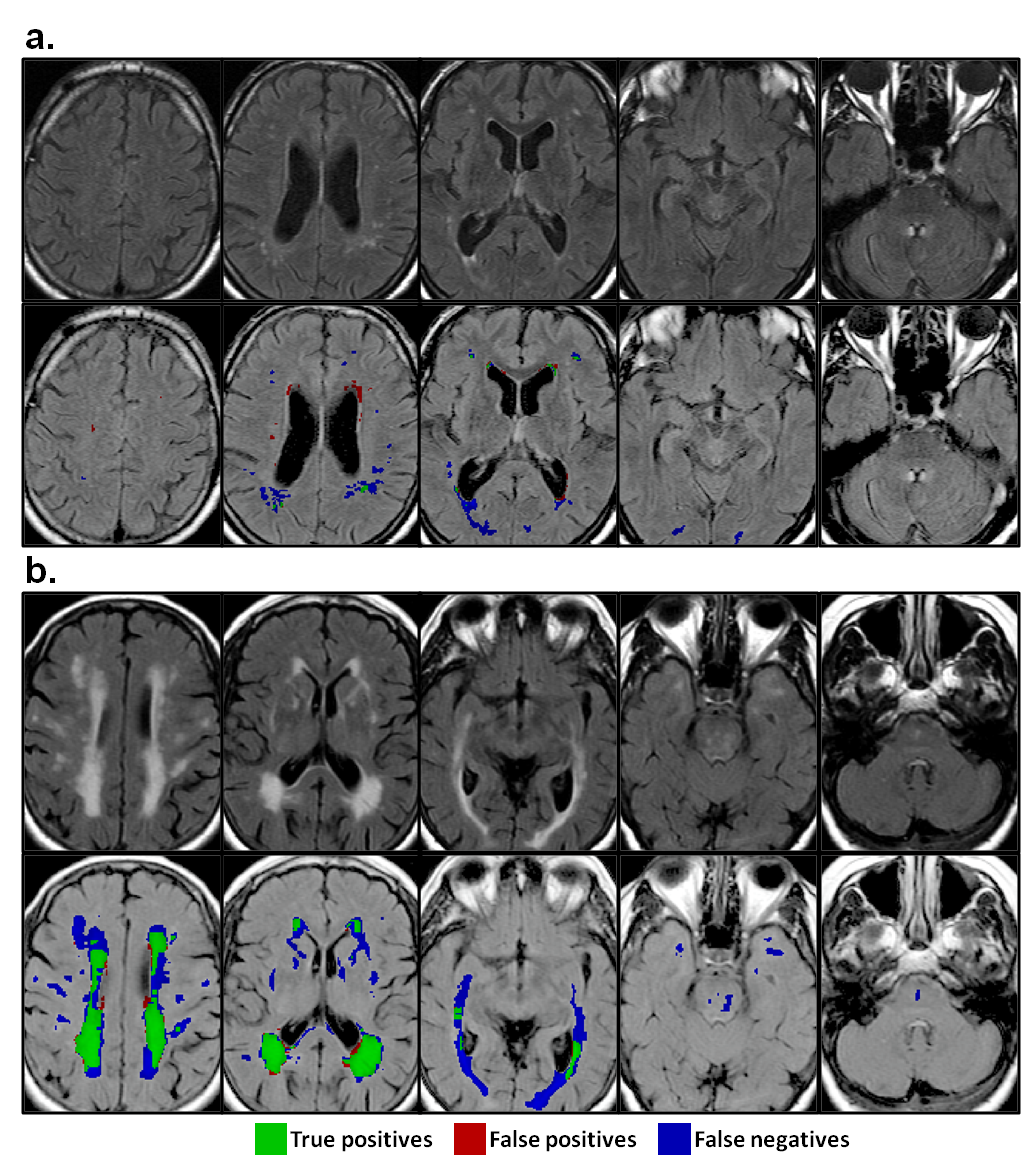
**

Figure S1.1 – Illustration of Freesurfer results.

SI being sensitive to total lesion load, dataset was restricted to subjects with a total lesion load between 10 and 80 cm^3^. The subjects with the highest and the lowest SI are displayed. a. Subject with lowest performance. (Reference volume: 10.5 mL; Freesurfer volume: 4.2 mL; SI=0.19). b. Subject with highest performance (Reference volume: 72.6 mL; Freesurfer volume: 34.8 mL; SI=0.61).


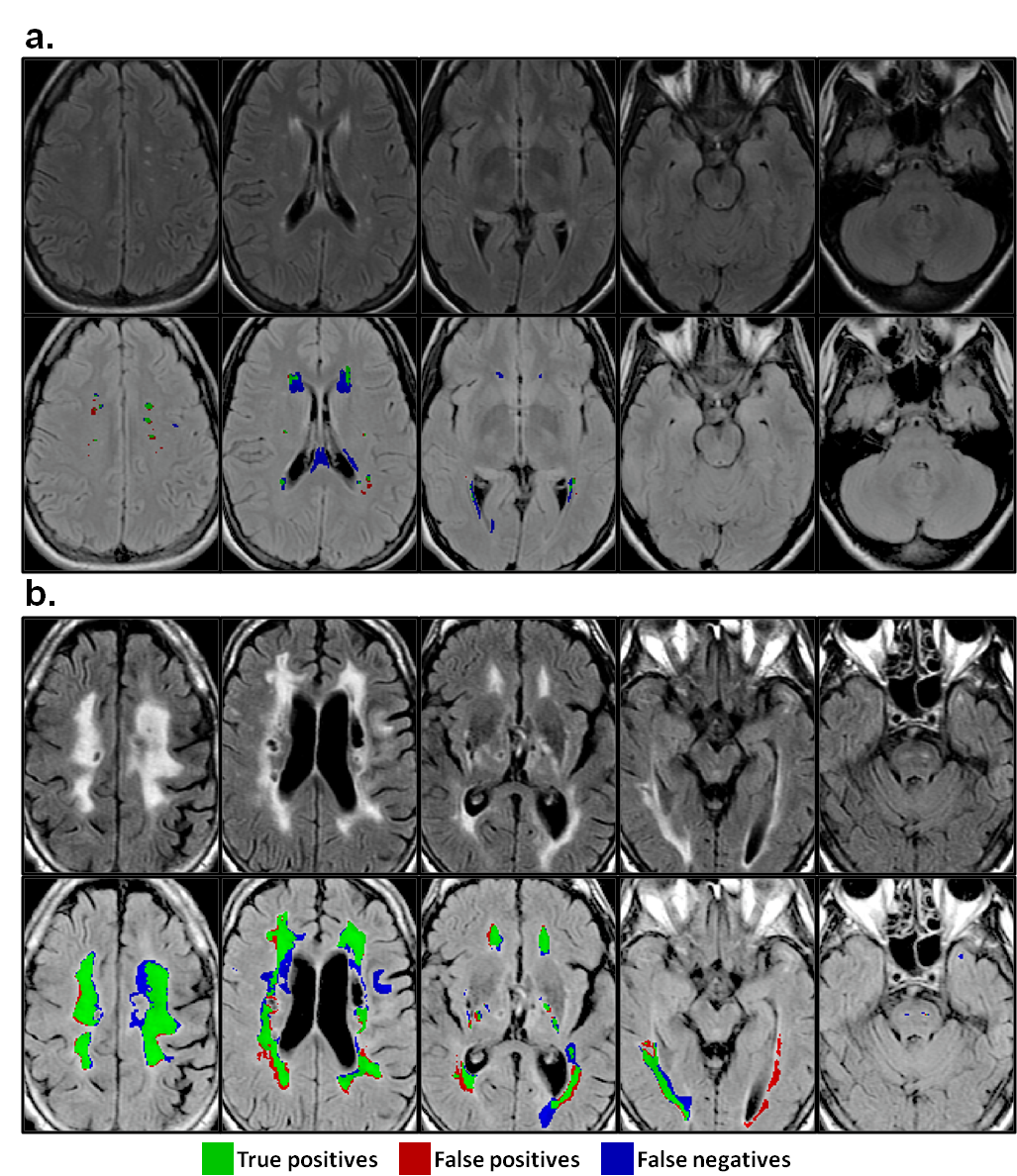


Figure S1.2 - Illustration of Thresholding results.

SI being sensitive to total lesion load, dataset was restricted to subjects with a total lesion load between 10 and 80 cm^3^. The subjects with the highest and the lowest SI are displayed. a. Subject with lowest performance. (Reference volume: 12.7 mL; Threshold volume: 4.2 mL; SI=0.36). b. Subject with highest performance (Reference volume: 69.9 mL; Threshold volume: 60.0 mL; SI=0.78).

**
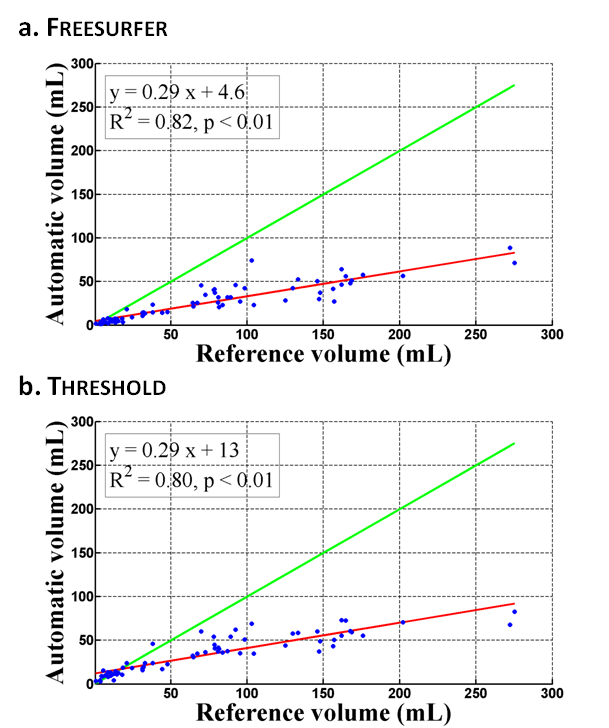
**

Figure S1.3 – Regression analyses for Freesurfer and Threshold
