# Supplementary material for: Contrast-Based Fully Automatic Segmentation of White Matter Hyperintensities: Method and Validation
Source: PLoS One. 2012 Nov 12;7(11):e48953. doi: 10.1371/journal.pone.0048953 (PMC3495958; doi:10.1371/journal.pone.0048953)
Supplement: File S2 — Results of supervised methods for all feature vectors and all test/training sets. (DOC) [file pone.0048953.s005.doc]

**Supporting information S2:**

**Results of supervised methods for all feature vectors and all test/training sets**

|  | | |  |  | **Regression analysis** | | | **Bland and Altman** | |
| --- | --- | --- | --- | --- | --- | --- | --- | --- | --- |
|  | | | **Mean SI (SD)** | **ICC** | **Slope** | **y-intercept** | **R^2^** | **Bias** | **95% limits** |
| **kNN** | **Test set 1** | **FV A** | 0.71 (0.19) | 0.90 | 0.79 | 29 | 0.83 | 11 | [-44 66] |
|  |  | **FV B** | 0.71 (0.18) | 0.90 | 0.77 | 27 | 0.83 | 8.3 | [-47 64] |
|  |  | **FV C** | 0.65 (0.16)* | 0.92 | 0.85 | 22 | 0.86 | 8.9 | [-41 59] |
|  |  | **FV D** | 0.65 (0.16)* | 0.92 | 0.84 | 21 | 0.86 | 7.3 | [-43 58] |
|  | **Test set 2** | **FV A** | 0.63 (0.22)* | 0.87 | 0.73 | 19 | 0.79 | 0.3 | [-56 57] |
|  |  | **FV B** | 0.62 (0.22)* | 0.87 | 0.71 | 19 | 0.79 | -0.2 | [-58 58] |
|  |  | **FV C** | 0.51 (0.27)* | 0.91 | 0.93 | 5.6 | 0.82 | 1.3 | [-53 55] |
|  |  | **FV D** | 0.51 (0.27)* | 0.91 | 0.92 | 5.8 | 0.82 | 0.7 | [-54 55] |
|  | **Test set 3** | **FV A** | 0.70 (0.19) | 0.91 | 0.75 | 22 | 0.86 | 3.0 | [-49 55] |
|  |  | **FV B** | 0.69 (0.20) | 0.91 | 0.75 | 21 | 0.86 | 1.9 | [-51 55] |
|  |  | **FV C** | 0.62 (0.19)* | 0.94 | 0.91 | 14 | 0.88 | 7.1 | [-40 54] |
|  |  | **FV D** | 0.62 (0.19)* | 0.94 | 0.90 | 14 | 0.88 | 5.7 | [-42 53] |
| **SVM** | **Test set 1** | **FV A** | 0.70 (0.21) | 0.89 | 0.74 | 29 | 0.82 | 6.8 | [-51 65] |
|  |  | **FV B** | 0.69 (0.19) | 0.89 | 0.74 | 30 | 0.83 | 8.7 | [-48 65] |
|  |  | **FV C** | 0.72 (0.16) | 0.89 | 0.68 | 17 | 0.88 | -10 | [-65 44] |
|  |  | **FV D** | 0.69 (0.18) | 0.88 | 0.67 | 21 | 0.87 | -7.3 | [-63 48] |
|  | **Test set 2** | **FV A** | 0.66 (0.22) | 0.88 | 0.77 | 24 | 0.80 | 9.0 | [-47 65] |
|  |  | **FV B** | 0.65 (0.22)* | 0.88 | 0.75 | 21 | 0.81 | 4.3 | [-51 59] |
|  |  | **FV C** | 0.67 (0.18) | 0.92 | 0.78 | 9.4 | 0.87 | -5.1 | [-51 41] |
|  |  | **FV D** | 0.47 (0.26)* | 0.90 | 0.85 | 4.4 | 0.80 | -5.7 | [-61 49] |
|  | **Test set 3** | **FV A** | 0.70 (0.19) | 0.92 | 0.77 | 22 | 0.88 | 4.3 | [-45 54] |
|  |  | **FV B** | 0.71 (0.18) | 0.92 | 0.77 | 18 | 0.88 | -0.2 | [-49 49] |
|  |  | **FV C** | 0.70 (0.19) | 0.94 | 0.80 | 15 | 0.90 | -0.9 | [-45 43] |
|  |  | **FV D** | 0.56 (0.20)* | 0.93 | 0.91 | 11 | 0.86 | 4.5 | [-47 56] |

Table S2.1 – Evaluation of different sub-cases for supervised methods

**
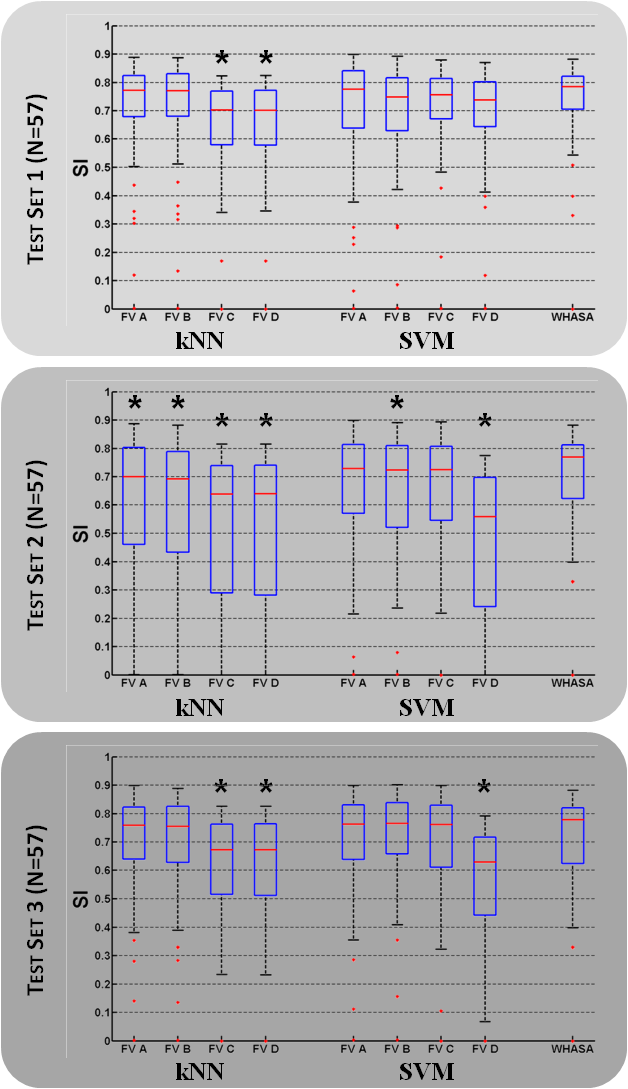
**

Figure S2.1 – SI distribution for different feature vectors

and different training/test sets.

*: significantly lower than WHASA methods (one-tailed t-test, p<0.05)
